# Alisol A 24-Acetate and Alisol B 23-Acetate Induced Autophagy Mediates Apoptosis and Nephrotoxicity in Human Renal Proximal Tubular Cells

**DOI:** 10.3389/fphar.2017.00172

**Published:** 2017-03-31

**Authors:** Chunfei Wang, Liang Feng, Liang Ma, Haifeng Chen, Xiaobin Tan, Xuefeng Hou, Jie Song, Li Cui, Dan Liu, Juan Chen, Nan Yang, Jing Wang, Ying Liu, Bingjie Zhao, Gang Wang, Yuanli Zhou, Xiaobin Jia

**Affiliations:** ^1^Key Laboratory of New Drug Delivery System of Chinese Materia Medica, Jiangsu Province Academy of Traditional Chinese MedicineNanjing, China; ^2^School of Pharmacy, Anhui University of Chinese MedicineHefei, China; ^3^Faculty of Health Sciences, University of MacauMacau, China; ^4^School of Pharmacy, Nanjing University of Chinese MedicineNanjing, China; ^5^School of Pharmaceutical Sciences, Xiamen UniversityXiamen, China

**Keywords:** alisol A 24-acetate, alisol B 23-acetate, autophagy, apoptosis, nephrotoxicity

## Abstract

Two natural compounds alisol A 24-acetate (24A) and alisol B 23-acetate (23B) are abundant in *Rhizoma alismatis.* In the present study, we evaluated the induction of 24A and 23B on apoptosis and possible nephrotoxicity of human renal proximal tubular (HK-2) cells by activating autophagy and also explored its regulation on PI3K/Akt/mTOR signaling pathway. Presently, Clusterin, Kim-1, and TFF-3 were considered to be new bioindicators of nephrotoxicity. Interestingly, the protein expression and mRNA levels of Clusterin, Kim-1 and TFF-3 could be significantly increased by 23B and 24A *in vivo* and *in vitro*. Furthermore, cell apoptosis could be triggered by 23B and 24A via significantly decreasing the protein expression and mRNA levels of Bcl-2 and Bcl-xl. Autophagy of HK-2 cells could be induced by both 23B and 24A via significantly enhancing the ratio of LC3II/LC3I, the protein expression of Beclin-1 as well as the mRNA levels of LC3 and Beclin-1. Meanwhile, PI3K/Akt/mTOR signaling pathway could be inhibited by these two compounds. An autophagy inhibitor, 3-methyladenine, could partially reverse cell viability and conversely change the ratio of LC3II/LC3I and the protein expression of Bcl-2 and Kim-1. Thus this study helped to understand that 23B and 24A induced autophagy resulted in apoptosis and nephrotoxicity through inhibiting PI3K/Akt/mTOR signaling pathway, facilitating further studies for nephrotoxicity induced by these two compounds and could be beneficial for safe use of *Rhizoma alismatis* in clinic.

## Introduction

Nephrotoxicity, an irreversible injury in renal, was caused by drugs, foods or other factors. Drug-induced kidney injury has become a major cause of nephrotoxicity, which attracted high attentions of researchers. Chinese herbs, described as an artificial intelligence of healthcare practices with a long history of use, were largely unregulated without registration, monitor or verification (Ikram et al., 2015; Stegelmeier et al., 2015). Chinese-herb nephropathy (CHN), a progressive renal interstitial fibrosis, was initially reported after intaking nephrotoxic components from Chinese herbs (Debelle et al., 2003). Nephrotoxic drugs could cause direct toxicity on renal function and this toxicity might depend on the clinical context involved (Finlay et al., 2013). Although relevant studies have proved that about 50 Chinese herbs could lead to nephrotoxicity including *Aristolochia debilis Sieb. et Zucc.* (Tsai et al., 2013), *Tripterygium wilfordii Hook. f.* (Li X.X. et al., 2015), *A. manshuriensis Kom.* (Ding et al., 2005), and so on. However, there was very limited information on nephrotoxicity of commonly used Chinese herbs.

Autophagy was a highly conserved physiological process involved in removing damaged or aged biological macromolecules and organelles from the cytoplasm (Cui et al., 2015). Autophagy played an important role in kidney as a double-edged sword. It could either be protective and hence contribute to survival, or promote death by non-apoptotic or apoptotic pathways (Thévenod and Lee, 2015). Recent studies showed that autophagy was a complicated process in the kidney including a protective effect by controlling autophagy within a certain range or a damage leading to nephrotoxicity by over expression (Shao et al., 2014; Takabatake et al., 2014; Wang and Choi, 2014). Currently, the role of autophagy in the pathogenesis of nephrotoxicity remains unclear.

**FIGURE 1:**
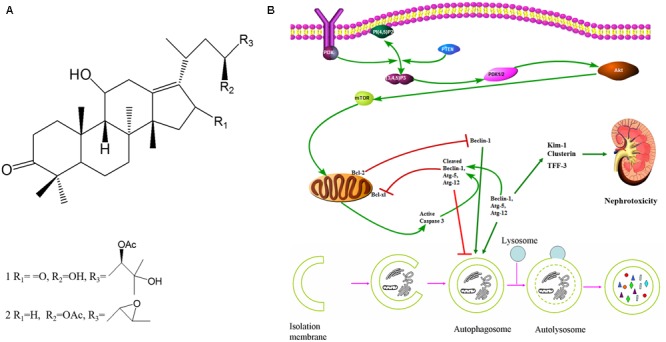
**23B and 24A induced autophagy regulated nephrotoxicity and apoptosis through PI3K/Akt/mTOR signaling pathway. (A)** Chemical structure of 24A (1) and 23B (2). (Alisol A 24-acetate: C_32_H_52_O_6_, molecular weight = 532.75; Alisol B 23-acetate: C_32_H_50_O_5_, molecular weight = 514.74). **(B)** The potential mechanism of autophagy regulated nephrotoxicity and apoptosis through PI3K/Akt/mTOR signaling pathway.

*Rhizoma alismatis* (RA), the dried rhizomes of *Alisma orientalis (Sam.) Juzep.*, known as “Zexie” in Chinese, has been commonly used for treating a wide range of ailments (Li and Qu, 2012; Han et al., 2013; Liu et al., 2013; Xu et al., 2013; Chen et al., 2014; Feng et al., 2014; Song et al., 2014; Tian et al., 2014). Although Alisol C, 23-epoxy-alisol B and Alisol O were identified as nephrotoxic components from RA, the controversy of RA nephrotoxicity was still unsettled (Zhao et al., 2011). Recently, Alisol B 23-acetate, Alisol A 24-acetate, and Alisol B were identified as novel inducers of autophagy, with Alisol B being the most potent natural component in RA (Law et al., 2010). RA inhibitory effects on OP9 cells are mediated autophagy by decreasing the expression of autophagy-related proteins, including Beclin-1 (Park et al., 2014). Consequently, 23B and 24A (**Figure 1A**) might take part in the formation of autophagy contributing to nephrotoxicity.

Herein, we conducted this investigation to explore: (1) the nephrotoxicity of 23B and 24A *in vitro* and *in vivo*; (2) the regulation of 23B and 24A on apoptosis and autophagy of HK-2 cells; (3) whether the possible nephrotoxicity and apoptosis of 23B and 24A was caused by autophagy; (4) this autophagy of HK-2 cells might be associated with the regulation of 23B and 24A on PI3K/Akt/mTOR signaling pathway (**Figure 1B**).

## Materials and Methods

### Chemicals and Materials

Trypsin was provided by KeyGEN Biotech Co., Ltd (Nanjing, China). Dulbecco’s modified eagle medium (DMEM)-F12 (1:1) medium with high-glucose and fetal bovine serum (FBS) were purchased from Gibco/BRL (Grand Island, New York, NY, USA). 3-4,5-dimethyl-2-thiazolyl)-2,5- diphenyl-2-H-tetrazolium bromide (MTT), dimethylsulfoxide (DMSO), and 3-methyl adenine (3-MA) were provided by Sigma Chemical (St. Louis, MO, USA). Alisol A 24-acetate (24A) and Alisol B 23-acetate (23B; purity ≥95%) were purchased from Tianjin Evans Science and Technology Co., Ltd (Tianjin, China). Primary and second antibodies against LC3, Beclin-1, Bcl-2, Bcl-xl, Clusterin, Kim-1, TFF-3 and β-actin were provided by Santa Cruz Biotechnology, Inc. (Dallas, TX, USA). Hematoxylin-eosin staining solution was offered by Shanghai Yuanmu Biotechnology Co., Ltd (Shanghai, China). EliVision plus and DAB kits were offered by MXB Biological Technology Co., Ltd (Fuzhou, Fujian, China). Monopotassium phosphate, disodium hydrogen phosphate, sodium chloride, and potassium chloride were obtained from Nanjing Nanao Science and Technology Co., Ltd (Nanjing, China).

### Animal Experiments

All animal procedures were approved by the Institutional Animal Care and Use Committee of Jiangsu Provincial Academy of Chinese Medicine in accordance with published National Institutes of Health guidelines. Thirty female Sprague Dawley (SD) rats (180 ± 20 g) from SLAC experimental animals Co., Ltd (Shanghai, China) were used. Before the experiment, rats were normally fed with diet and distilled water *ad libitum*. Rats were randomly divided into 3 groups with 10 for each group: Control group rats were gavaged with sodium chloride (10 mL/kg/day), 23B and 24A group rats were gavaged with 23B (0.4 g/kg/day) and 24A (0.5 g/kg/day), respectively, for 6 months according to our preliminary experiment (According to clinic prescription, the maximum dosage of RA is 45 g/kg/day for healthy adults. Then animal dosages were calculated with the content of 23B and 24A in Fujian RA determined by us).

### H&E Staining

After rats being sacrificed, the kidneys were removed and immediately fixed in formalin solution. The kidneys were embedded in paraffin and cut into 5 μm thick sections and stained with hematoxylin-eosin (H&E) (Kandemir et al., 2015). The kidney sections were observed under the IX51 microscope (Olympus Corporation, Japan).

### Cell Culture

Human renal proximal tubular cell line (HK-2) was purchased from Shanghai Institute of Biochemistry and Cell Biology (Shanghai, China). Cells were cultured as described previously (Gong and Hou, 2016), in high-glucose DMEM-F12 (1:1) medium with 10% FBS, containing penicillin (80 units/mL) and streptomycin (0.08 mg/mL). Then cells were placed in an incubator at 37°C with 5% CO_2_ and medium should be replaced every other day. After having grown to 90% confluence, cells should be digested by 0.25% trypsin-0.02% EDTA for the passage.

### Analysis of Cell Viability by MTT

After 90% confluence, the cells were seeded into 96-well plates (5 × 10^3^ cells/well, 100 μL). Cells were treated with different concentrations of 24A (768, 384, 192, 96, 48, 24, 12, 6, 3 μM) and 23B (960, 480, 240, 120, 60, 30, 15, 7.5, 3.25 μM) for 24 h. Then, 100 μL MTT stock solution (5.0 mg/mL) was added to each well for 4 h to form purple crystal formazan. At the end of incubation, DMSO of 100 μL was added for 10 min microvibration after removing medium. The absorbance was measured at 570 nm on a microplate reader (Thermo, New York, NY, USA).

### Flow Cytometry

Cell apoptosis was analyzed by Annexin V and propidium iodide (PI) staining using flow cytometry (Peng et al., 2015), with the Annexin V-FITC/PI assay kit (Nanjing KeyGen Biotech, Nanjing, China) according to the manufacturer’s instructions. FITC Annexin V and PI were 5 μL, respectively, which was added in sequence for cells staining after being trypsinized, centrifuged and resuspended. After incubation with staining solution (annexin V/PI = 1:2) for 20 min in the dark at room temperature. Fluorescence-activated cell sorting (FACS) analysis was immediately performed using a flow cytometer (BD Biosciences, San Jose, CA, USA).

### Transmission Electron Microscopy (TEM)

HK-2 cells were fixed and embedded as described previously (Lu et al., 2014). Cells were fixed with 5% glutaraldehyde followed by PBS (pH = 7.4) washing. Then cells were post-fixed in 1% osmic acid and dyed with 2% uranyl acetate. Sequentially, cells were dehydrated by 50, 70, 90, and 100% acetone and embedded in EPON812 resin. Samples were analyzed using the JEM-1010 transmission electron microscope (JEOL, Japan) at a voltage of 100 kV.

### Immunofluorescence Analysis

LC3 immunofluorescence analysis was conducted as previous study (Kang et al., 2014). HK-2 cells were grown on glass coverslips in six-well plates to 60% confluence and then treated with 23B (15 μM) and 24A (6 μM) for 24 h. After being fixed with 4% paraformaldehyde, cells were permeabilized with PBS containing 1% Triton-100. Rabbit antibody (1:100) and goat anti-rabbit IgG-FITC (1:400) was, respectively, used as the primary and secondary antibody. The nuclei were colabeled with DAPI solution. The images were collected using an IX51 fluorescence microscope (Olympus, Japan). The percentage of HK-2 cells showing accumulation of LC3 puncta was quantified by Image-Pro Plus picture analysis software (Media Cybernetics, Rockville, MD, USA).

### Immunocytochemistry Assay

Cells were plated on coverslip at initial densities of 5 × 10^5^ cells/ piece and incubated for 24 h prior to the addition of 23B (15 μM) and 24A (6 μM). Then the cells were trypsinized, washed with PBS and fixed with fresh 4% paraformaldehyde solution. To retrieve antigens, the sections were heated in 10 mM sodium citrate buffer solution (pH = 6.0) for 20 min. According to endogenous peroxidase, slides were incubated in 0.3% H_2_O_2_ of methanol to reduce non-specific background staining. Sequentially, cells were boiled in citrate buffer solution for 10 min. They were cooled and then washed by PBS before the application of blocking serum. Primary antibody (1:200) were incubated with cells and then probed with secondary antibody. Elivison two-step method was performed for the immunocytochemistry staining. The pictures were collected by microscope. The positive expression was calculated by Image-Pro Plus picture analysis software (Media Cybernetics, Rockville, MD, USA).

### Western Blotting

Levels of protein in cells or kidneys were determined with sodium dodecyl sulfate-polyacrylamide gel electrophoresis (SDS-PAGE) combined with western blotting analysis. Briefly, proteins were extracted from tissue homogenates or cells and an equal amount of total protein was separated by 10% SDS-PAGE and then transferred from the SDS-PAGE gel to PVDF membrane (Millipore, USA). After being blocked with 5% BSA in tris-buffered saline Tween-20 (TBST), membranes were incubated with the primary antibodies at a dilution of 1:500 overnight. Subsequently, membrane was washed (three times, 5 min), and incubated with secondary antibody (1:1000) at 37°C for 30 min. The blots were visualized with ECL-Plus reagent (Santa Cruz, USA) and analyzed with Image Pro Plus picture analysis software (Media Cybernetics, Rockville, MD, USA). β-actin was used as loading control.

### Quantitative PCR

Total RNA of HK-2 cells were extracted by TRIzol reagent (Invitrogen, USA). Then it was reverse transcribed with a SuperScript III First-Strand Synthesis System for quantitative polymerase chain reaction (q-PCR) following manufacturer’s indications (Springen, Nanjing, China). Primer sequences of GAPDH, LC3, Beclin-1, Clusterin, Kim-1, TFF-3, Bcl-2, and Bcl-xl were shown in **Table 1**. GAPDH was used as the internal reference. q-PCR was achieved by an ABI 7900 sequence detector (Life Technologies, Carlsbad, CA, USA) with the SYBR Green method and d(N) six random hexamer with primers purchased from Invitrogen (Carlsbad, CA, USA). Sequentially, the thermocycling parameters were 95°C for 10 min, 40 cycles of 95°C for 15 s and 60°C for 1 min. Samples were run in triplicate and were normalized to 18S RNA. Fold changes were determined using the DDCt method.

**Table 1:** Sequences of primers used for mRNA detection.

Indicator	Primer sequences (5′-3′)	
GAPDH	Sense primer	CATCTTCTTTTGCGTCGCCA
	Antisense primer	TTAAAAGCAGCCCTGGTGACC
LC3	Sense primer	AGTGCCTGTGTTGTTACGGA
	Antisense primer	GCAGAAGGGAGTGTGTCTGA
Beclin-1	Sense primer	AATGACTTTTTTCCTTAGGGGG
	Antisense primer	GTGGCTTTTGTGGATTTTTTCT
TFF-3	Sense primer	CCAAGCAAACAATCCAGAGCA
	Antisense primer	GCTCAGGACTCGCTTCATGG
Kim-1	Sense primer	TGGCAGATTCTGTAGCTGGTT
	Antisense primer	AGAGAACATGAGCCTCTATTCCA
Clusterin	Sense primer	CCAATCAGGGAAGTAAGTACGTC
	Antisense primer	CTTGCGCTCTTCGTTTGTTTT
Bcl-xl	Sense primer	GAGCTGGTGGTTGACTTTCTC
	Antisense primer	TCCATCTCCGATTCAGTCCCT
Bcl-2	Sense primer	AATATCCAATCCTGTGCTGCTA
	Antisense primer	GTCCACGTTCTTCATTGTTACTTC

### Statistical Analysis

SPSS 16.0 software was used to calculate the statistical differences among different groups by one-way ANOVA followed by Tukey’s test. *P* values smaller than 0.05 were considered as statistically significant.

## Results

### 23B and 24A Affect Cell Viability and Activate Cell Apoptosis

**FIGURE 2:**
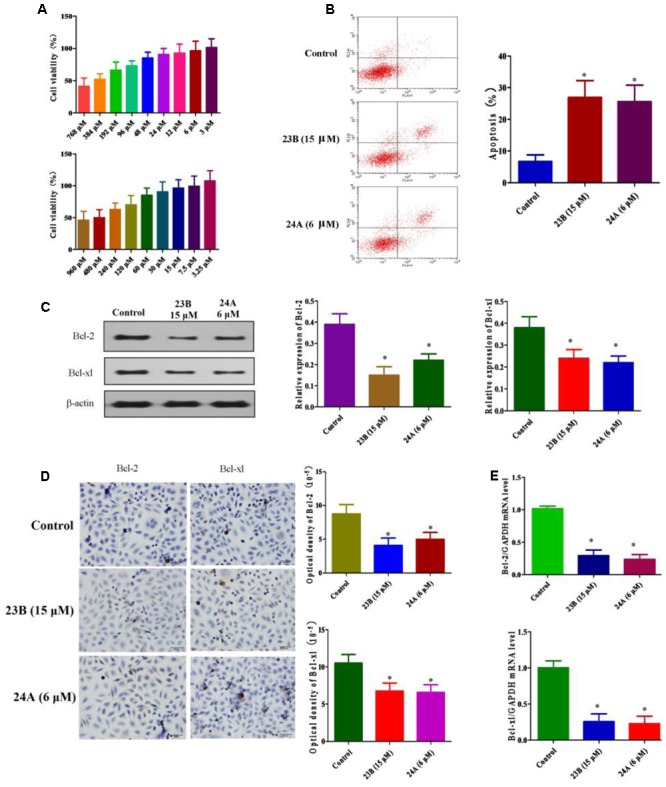
**Apoptosis related protein expression in HK-2 cells treatment with 23B and 24A. (A)** Cell viability of 23B and 24A in different concentration. Data are expressed as means ± SD, *n* = 6. **(B)** Cells were treated with 23B and 24A and cell apoptosis were analyzed by flow cytometry. **(C)** Cells were treated with 23B and 24A and protein levels were analyzed by western blotting. Protein expressions were semi-quantified by densitometry analysis. **(D)** Immunocytochemistry assay for protein expression in HK-2 cells. Protein expressions were semi-quantified by densitometry analysis. **(E)** The mRNA levels of Bcl-2 and Bcl-xl. All experiments were repeated at least three times. Data were represented as mean ± SD (*n* = 3). ^∗^*p* < 0.05, control group vs. 23B group, control group vs. 24A group.

MTT assay was used to assess cell viability for screening the optimal drug concentration of 23B and 24A to HK-2 cells. As shown in **Figure 2A**, 15 μM 23B (cell viability: 96.69 ± 14.74%) and 6 μM 24A (cell viability: 96.20 ± 13.94%) were chosen for further experiments with cell viabilities were both higher than 95%.

Cell apoptosis was evaluated by the Annexin V and PI double stain with flow cytometry. As depicted in **Figure 2B**, the treatment with 23B (15 μM) and 24A (6 μM) significantly increased HK-2 cells apoptosis (*P* < 0.05), compared with control blank group. Additionally, to further testify cell apoptosis induced by 23B and 24A, western blot analysis and immunocytochemistry assay were used to determine the protein expression of Bcl-2 and Bcl-xl in HK-2 cells (**Figures 2C,D**). Compared with control blank group, the expression of anti-apoptotic Bcl-2 and Bcl-xl were significantly decreased by intervening with 23B and 24A (*P* < 0.05). Moreover, the mRNA levels of Bcl-2 and Bcl-xl were significantly decreased (*P* < 0.05) in contrast with control blank group (**Figure 2E**). Thus the results indicated that 23B and 24A could trigger apoptosis in HK-2 cells via down-regulating the expression of Bcl-2 and Bcl-xl.

### Speculation of Nephrotoxicity Induced by 23B and 24A *In vitro* and *In vivo*

**FIGURE 3:**
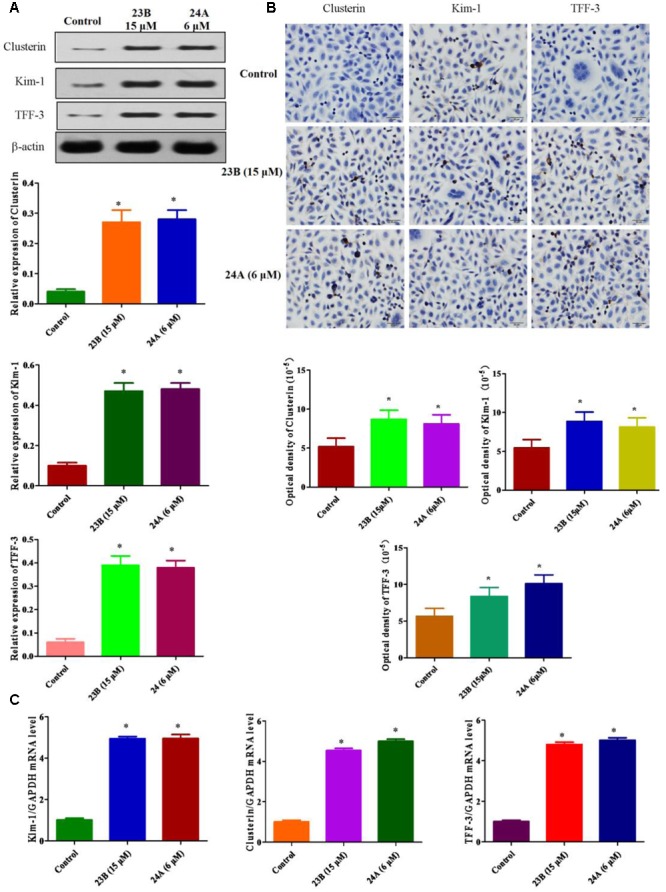
**Determination of nephrotoxicity related protein expression basing on HK-2 cells. (A)** Cells were treated with 23B and 24A and protein levels were analyzed by western blotting. Protein expressions were semi-quantified by densitometry analysis. **(B)** Immunocytochemistry assay for protein expression in HK-2 cells. Protein expressions were semi-quantified by densitometry analysis. **(C)** The mRNA levels of Kim-1, Clusterin and TFF-3. Data are expressed as means ± SD, *n* = 3. ^∗^*p* < 0.05, control group vs. 23B group, control group vs. 24A group.

In this study, we explored whether nephrotoxicity was induced by 23B and 24A *in vitro* and *in vivo*. *In vitro*, we used HK-2 cells to explain 23B and 24A induced nephrotoxicity, western blot analysis and immunocytochemistry assay were used to determine the protein expressions of Kim-1, Clusterin and TFF-3 in HK-2 cells (**Figures 3A,B**). Compared with control blank group, the expressions of Kim-1, Clusterin and TFF-3 were significantly increased by treating with 23B and 24A (*P* < 0.05). To further evaluate nephrotoxicity, q-PCR was also used to detect the mRNA level of Kim-1, Clusterin and TFF-3. As described in **Figure 3C**, the mRNA levels of Kim-1, Clusterin and TFF-3 were significantly increased by exposure to 23B and 24A as compared with control blank group (*P* < 0.05).

**FIGURE 4:**
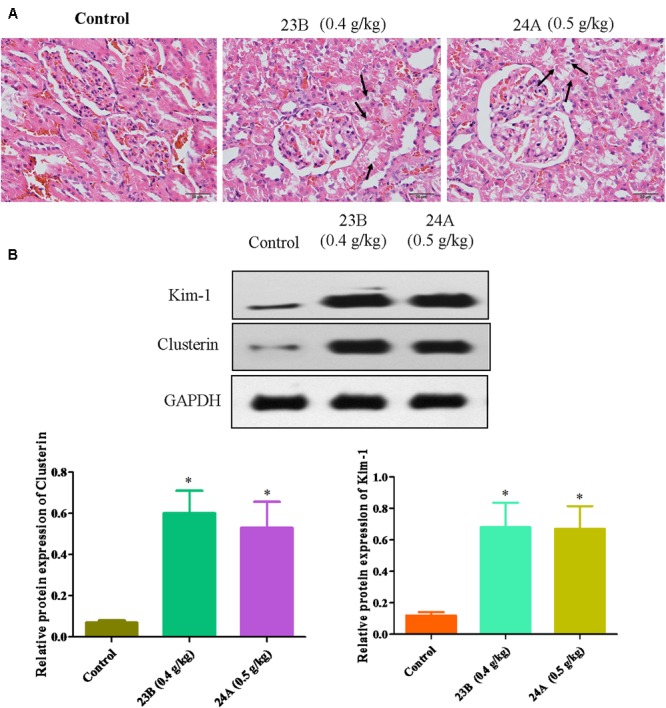
**Exploration of 23B and 24A induced nephrotoxicity in rats. (A)** HE staining of rat kidney after 6 months drug treating, pathological changes were shown by black arrows. **(B)** Protein levels in rat kidney were analyzed by western blotting. Protein expressions were semi-quantified by densitometry analysis. Data are expressed as means ± SD, *n* = 3. ^∗^*p* < 0.05, control group vs. 23B group, control group vs. 24A group.

*In vivo*, pathological changes of kidneys from rats were identified. As shown in **Figure 4A**, interstitial inflammation, renal tubular epithelial cell exfoliation and morphological changes were observed in 23B or 24A-treated rat kidneys. Western blot was also used to determine the protein expressions of Kim-1, Clusterin and TFF-3 in rat kidneys. We also found that the protein expressions of Kim-1, Clusterin and TFF-3 were significantly increased by treatment of 23B and 24A in rat kidneys, comparing with control blank group (*P* < 0.05, **Figure 4B**).

Kim-1, Clusterin and TFF-3 were important biomarkers in drug induced nephrotoxicity accepted by Food and Drug Administration (FDA), the European Agency for the Evaluation of Medicinal Products (EMEA) and Critical Path Institute, Predictive Safety Testing Consortium (C-Path PSTC) (Yu et al., 2013). It is reported that they could protect HK-2 cells from apoptosis in drug induced nephrotoxicity for early detection (Khan and Pandey, 2014; Mohamed et al., 2015). Above all, basing on the up-regulating expressions of Kim-1, Clusterin and TFF-3, we speculated that 23B and 24A could trigger nephrotoxicity accompanied with cell apoptosis.

### 23B and 24A Induced Autophagy Formation of HK-2 Cells

**FIGURE 5:**
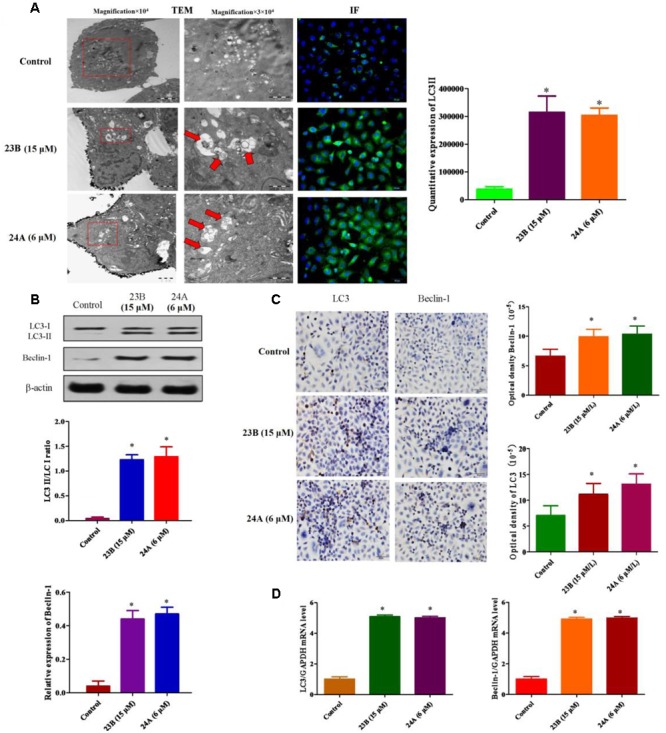
**23B and 24A promoted the formation of autophagy. (A)** 23B and 24A induce the formation of autophagy. The ultrastructures of HK-2 cells were analyzed by TEM and immunofluorescent staining of LC3II. Autophagosomes, autolysosomes or engulfed organelles was shown by red arrows. LC3II expression was semi-quantified by densitometry analysis. **(B)** Cells were treated with 23B and 24A and protein levels were analyzed by western blotting. Protein expressions were semi-quantified by densitometry analysis. **(C)** Immunocytochemistry assay for protein expression in HK-2 cells. Protein expressions were semi-quantified by densitometry analysis. **(D)** The mRNA levels of LC3 and Beclin-1. All experiments were repeated at least three times. Data were represented as mean ± SD (*n* = 3). ^∗^*p* < 0.05, control group vs. 23B group, control group vs. 24A group.

23B and 24A were identified as inducers of autophagy in previous study (Law et al., 2010). The ultrastructures of HK-2 cells were analyzed by TEM to show the formation of autophagy. Numerous autophagosomes characterized by double membrane structures were observed in cells treated with 23B (15 μM) and 24A (6 μM) and autophagic vacuoles containing degraded organelles were also observed (**Figure 5A**). In addition, **Figure 5A** depicted immunofluorescence images of HK-2 cells incubated with 23B and 24A. Significantly higher green fluorescence was visible inside cells after 24 h incubation with 23B (15 μM) and 24A (6 μM), indicating the increase of LC3II. Quantitative expression of LC3II was shown in **Figure 5A**, a significant raising in the amount of LC3II was shown after 24 h treatment with 23B and 24A (*P* < 0.05). The results suggested that 23B and 24A could induce autophagy of HK-2 cells.

In order to further identify autophagy induced by 23B and 24A, western blot analysis and immunocytochemistry assay were also used. LC3II/LC3I ratio and Beclin-1 were chosen to illustrate the formation of autophagy. As shown in **Figures 5B,C**, the ratio of LC3II/LC3I and the expression of Beclin-1 in cells treated with 23B (15 μM) and 24A (6 μM) were significantly increased, respectively, (*P* < 0.05) comparing with control blank group. Besides, q-PCR was also used to evaluate autophagy induced by 23B and 24A. As is shown in the **Figure 5D**, in contrast with control blank group, the mRNA level of LC3 and Beclin-1 in 23B (15 μM) and 24A (6 μM) treated HK-2 cells were significantly increased, respectively, (*P* < 0.05). The results above further illustrated that 23B and 24A could induce autophagy in HK-2 cells via regulating the level of LC3 and Beclin-1.

### Activation of Autophagy Mediates Nephrotoxicity and Apoptosis through PI3K/AKT/mTOR Pathway in HK-2 Cells

**FIGURE 6:**
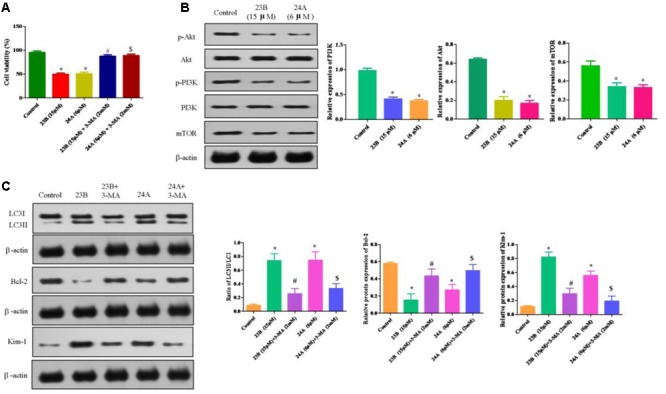
**Protein expression in 23B and 24A treated HK-2 cells. (A)** Cell viability of HK-2 cells incubated with autophagy inhibitor. Data are expressed as means ± SD, *n* = 6. ^∗^*p* < 0.05, control group vs. 23B group, control group vs. 24A group; ^#^*p* < 0.05, 23B group vs. 23B + 3-MA group; ^$^*p* < 0.05, 24A group vs. 24A + 3-MA group. **(B)** Cells were treated with 23B and 24A and protein levels were analyzed by western blotting. Protein expressions were semi-quantified by densitometry analysis. **(C)** 23B and 24A treated cells were co-incubated with 3-MA and protein levels were analyzed by western blotting. Protein expressions were semi-quantified by densitometry analysis. Data are expressed as means ± SD, *n* = 3. ^∗^*p* < 0.05, control group vs. 23B group, control group vs. 24A group; ^#^*p* < 0.05, 23B group vs. 23B + 3-MA group; ^$^*p* < 0.05, 24A group vs. 24A + 3-MA group.

To test whether nephrotoxicity and apoptosis were related to autophagy, 23B and 24A treated HK-2 cells were co-incubated for 48 h with 3-MA, a non-specific autophagy inhibitor. This treatment led to 49.65 ± 2.63 and 50.71 ± 3.47% in the viability of 23B and 24A-treated cells, while 87.30 ± 3.17 and 89.06 ± 3.03% in viability after co-incubated with 3-MA (**Figure 6A**). The results suggested that suppression of 23B and 24A induced autophagy with generic inhibitor could effectively reverse cell apoptosis.

The ratio of LC3II/LC3I was significantly decreased in HK-2 cells intervened by 23B and 3-MA or 24A and 3-MA through inhibiting the conversion of LC3II and further attenuating cell apoptosis by remarkably enhancing the protein expression of Bcl-2 (*P* < 0.05) while significantly reducing the protein expression of Kim-1 for the improvement of nephrotoxicity (*P* < 0.05) (**Figure 6B**), compared with that in 23B or 24A alone treated HK-2 cells. The results suggested that the generic autophagy inhibitor could effectively reverse cell apoptosis and nephrotoxicity via inhibiting 23B and 24A induced autophagy.

PI3K/Akt/mTOR, the classical autophagy signaling pathway, plays an important role in autophagy. Compared with control blank group, phosphorylation levels of PI3K, Akt and mTOR were significantly decreased after being treated with 23B or 24A (**Figure 6C**). Our results indicated that 23B or 24A induced autophagy could regulate nephrotoxicity and apoptosis through inhibiting PI3K/AKT/mTOR pathway in HK-2 cells, but the detailed mechanism still needs our further study and certification.

## Disscussion

Autophagy, as a name for cellular self-digestion, is a cellular pathway involved in protein or organelle degradation (Mizushima et al., 2008). Autophagy includes autophagosome and autolysosome while the formation of autophagosomes depend on several genes such as microtubule-associated protein 1 light chain 3 (LC3), Beclin-1 and autophagy-related genes (Zhao et al., 2015). LC3, associated with controlling of autophagosome elongation, is present in the LC3I under normal conditions, but recruited to membrane and interacts with phosphatidylethanolamine converting to LC3-II (Woo et al., 2016). Increased levels of LC3-II may be indicative of the formation of autophagy, which was consistent with our results. The complex, formed by connecting Beclin-1 with type III PI3K, adjusted other Atg protein to locate in autophagy precursor, which regulated the activity of autophagy (Wirth et al., 2013). Furthermore, the expression of Beclin-1 was up-regulated by stimulating the occurrence of autophagy.

Autophagy and apoptosis had a complex interplay, which can act in a coordinated and cooperative manner to induce cell death with autophagy blocking or facilitating execution of apoptotic cell death (Oral et al., 2016). Apoptosis is mainly characterized by internucleosomal DNA fragmentation and in most cases by activation of executioner/effector caspases, such as Bcl-2, which plays an important role in the initiation and maintenance of apoptosis (Melo-Lima et al., 2015). Bcl-2 and Bcl-xl are the main anti-apoptotic members in the Bcl-2 family. Interestingly, the Bcl-2 family exhibits the intercross properties in apoptosis and autophagy. Under basal conditions, Bcl-2 proteins were found to be constitutively bound to Beclin-1, and allowing only basal levels of autophagy to proceed (Oral et al., 2016). The dissociation of Beclin-1 from Bcl-2 could be an important event to allow autophagy while cleavage of Beclin-1 mediated by caspase might promote apoptosis (Morais et al., 2016). The functional relationship between autophagy and apoptosis is complex, and the relevant molecular mechanisms remain unclear. In several cases, autophagy is a stress adaptation that suppresses apoptosis and prevents cell death (Sureshbabu et al., 2015). Nevertheless, in our study, the results indicated that autophagy could promote apoptosis and accelerate cell death. 3-MA, an autophagy inhibitor, had an effectively inhibitory effect on autophagy to reverse cell apoptosis.

Phosphatidylinositol 3-kinase/protein kinase B/mammalian target of rapamycin (PI3K/Akt/mTOR) is involved with different cellular processes from cell growth or survival to cell death or apoptosis (Morgan et al., 2009). Phosphorylation of Akt up-regulated by the activation of PI3K and mTOR can integrate upstream activating signals through PI3K/Akt pathway leading to phosphorylate and inhibit autophagy (Polivka and Janku, 2014). Evidence that the inhibition of PI3K/Akt/mTOR signaling accelerates excessive autophagy that leads to apoptosis and the inhibition of mTORC1 may excite autophagy of damaged or toxic proteins leading to cellular death through mTORC2 activity on Akt (Heras-Sandoval et al., 2014). What’s more, 23B and 24A induced autophagy via inhibiting PI3K/Akt/mTOR signal pathway in our study.

Kidney is a typical organs exhibiting easily damage by xenobiotics. Autophagy is upregulated by hypoxia and oxidant injury, most of which are involved in the pathogenesis of nephrotoxicity (Kaushal and Shah, 2016). These days, Kim-1, clusterin and TFF-3 were widely recommended as novel biomarkers in nephrotoxicity which could protect cell apoptosis caused by kidney injury (Mizushima et al., 2008; Khan and Pandey, 2014). Kim-1 is a type one transmembrane glycoprotein that is not detectable in normal kidney tissue but highly up-regulated during acute kidney injury (Nogare et al., 2015; Yang et al., 2015). Clusterin is a glycoprotein with a slightly ubiquitous tissue distribution and an apparent involvement in biological processes (García-Martínez et al., 2012). Clusterin is not detectable in the healthy mature kidney, but its expression will be up-regulated in renal tubular injury and a variety of renal diseases (Li J.Y. et al., 2015). TFF-3, called intestinal trefoil factor or Itf, is a peptide predominantly along the gastrointestinal tract and in serum (Ge et al., 2015). An increase of TFF-3 may be secreted from renal tubular epithelial cells in damaged kidneys (Yu et al., 2010; Du et al., 2013). In the present study, 23B or 24A induced nephrotoxicity were evaluated with Kim-1, clusterin and TFF-3, which were novel biomarkers for fast and sensitive determination of nephrotoxicity and different from blood urea nitrogen (BUN) and serum creatinine (Scr).

**FIGURE 7:**
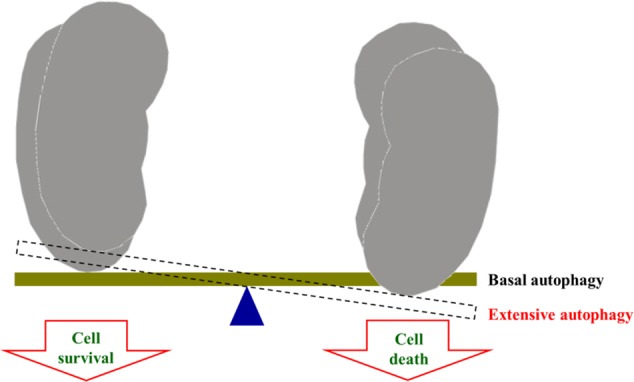
**The dual function of autophagy in kidney**.

More and more attentions have been paid to the safety of medicinal herbs in clinic. However, it has been influenced by many factors, such as body diathesis, irrational medication, long-term medication, high dose of medication, and so on. The most important reason is lacking awareness of drug-use safety. In addition, with the development of pharmaceutical technologies, the components in Traditional Chinese medicine preparations were purer and purer, so that it will increase the risk of toxicity. In this study, we found that 23B and 24A could induce nephrotoxicity, but there is no report about RA nephrotoxicity in clinic. This may be due to following reasons. First, 23B and 24A, main components in RA, could induce nephrotoxicity which is unequal to RA nephrotoxicity. Second, the content of RA is low in Traditional Chinese Medicine prescriptions. For example, the percentage of RA is 12% in Six Ingredient Rehmannia Pill (Liu Wei Di Huang Wan in Chinese). According to the instruction of Six Ingredient Rehmannia Pill, only 1.08 g RA has been taken by a health adult each day. What’s more, the contents of 23B and 24A in 1.08 g RA are too low to induce toxicity. Third, either RA or prescriptions containing RA don’t reach the toxic dose exposing in human body. Forth, basing on Network Toxicology, we think that there might be a certain threshold in autophagy regulation including protective function within the threshold as well as damages leading to nephrotoxicity by over expression of autophagy (**Figure 7**). However, this needs to be intensively studied. 23B and 24A are main components in RA, it can be accumulated in human body after a long use. There is no report of RA nephrotoxicity, but we should use it rational boost the sense of drug-use safety.

In this study, 23B or 24A triggered apoptosis and damaging HK-2 cells was proved. Moreover, autophagy inhibitor effectively reversed cell apoptosis and nephrotoxicity induced by 23B and 24A. Therefore, we surmised that this damage might be achieved by triggering autophagy in HK-2 cells via inhibition of PI3K/Akt/mTOR signaling pathway. However, a sort of mechanism existed between autophagy and nephrotoxicity still needs further study.

## Author Contributions

CW Analysis and interpretation of data, writing of the manuscript. LM Conception and design, acquisition of data. HC Acquisition of data, analysis and interpretation of data. GW Analysis and interpretation of data. BZ and YL Acquisition of data, analysis and interpretation of data, proof-reading of the manuscript. YZ, DL, and JW Development of methodology, revision of the manuscript. JC, NY and XH Technical support, analysis and interpretation of data. JS, LC and XT Conception and design, interpretation of data, revision of the manuscript. XJ and LF Conception and design, study supervision, revision of the manuscript. All authors read and approved the final manuscript.

## Conflict of Interest Statement

The authors declare that the research was conducted in the absence of any commercial or financial relationships that could be construed as a potential conflict of interest.
